# Guiding Principles for the Clinical Use and Selection of Microcatheters in Complex Coronary Interventions

**DOI:** 10.3389/fcvm.2022.724608

**Published:** 2022-03-09

**Authors:** Pravin K. Goel, Ankit Kumar Sahu, Sridhar Kasturi, Sanjeeb Roy, Nimit Shah, Prakashvir Parikh, Davinder S. Chadha

**Affiliations:** ^1^Department of Cardiology, Sanjay Gandhi Post Graduate Institute of Medical Sciences (SGPGI), Lucknow, India; ^2^Department of Cardiology, Sunshine Hospitals, Hyderabad, India; ^3^Department of Cardiology, Fortis Escorts Hospital, Jaipur, India; ^4^Department of Cardiology, Saifee Hospital, Mumbai, India; ^5^Department of Cardiology, Sir H.N. Reliance Foundation Hospital and Research Centre, Mumbai, India; ^6^Department of Cardiology, Dr. Jivraj Mehta Health Care, Ahmedabad, India; ^7^Department of Cardiology, SAL Hospital, Ahmedabad, India; ^8^Department of Cardiology, Vikram Hospital, Bengaluru, India

**Keywords:** microcatheter, complex percutaneous coronary interventions, selection criteria, single-lumen microcatheter, dual-lumen microcatheter

## Abstract

The use of microcatheters as a coronary interventional tool for a therapeutic approach to complex coronary interventions like bifurcation lesions, ostial location, tortuous anatomy, angled takeoffs, coronary calcification, and chronic total occlusion (CTO) percutaneous coronary intervention (PCI) is growing among cardiologists across the country. During the treatment of such complex lesions, microcatheters play an essential part of the tool kit with both single-lumen and double-lumen microcatheters (DLMs) having their specific niche areas. The selection of microcatheters involves a detailed understanding of the microcatheter specification, lesion anatomy, lesion location, vessel tortuosity and trajectory, and crossing techniques. The selection of appropriate crossing techniques with different microcatheters increases success rates of PCI, reduces procedural time and contrast use, and lowers the radiation. However, the use of microcatheters and their technicalities have not yet fully realized by many operators and their true scope has not been fully explored. This article discusses and summarizes the thoughts and key opinions of experts in this field.

## Introduction

Coronary lesions often exhibit certain features posed as challenging while performing a percutaneous coronary intervention (PCI) *viz*. thrombus, bifurcation, ostial lesion, calcification, and chronic total occlusions (CTOs) (1). The use of microcatheters as a coronary interventional tool assists in performing invasive endovascular procedures by serving a broad range of functions in these complex vessel anatomies.

The objective of this article is to formulate and deliberate on the recommendations for the selection of microcatheters and their utility in coronary interventional procedures. The discussion included broadly the following two topics:

- Understanding the significance of a microcatheter in the cardiac catheterization lab—which included a discussion on the types of microcatheter available in the Indian market and their significance in PCI procedures.
- Criteria for the selection of microcatheters—which included a discussion on the selection and preference of one microcatheter over another based on lesion/anatomy-specific criteria or hardware characteristics.

## Types of Microcatheter Available for Indian Patients Requiring PCI Procedures

### Single-Lumen Microcatheters

**Figure 1:**
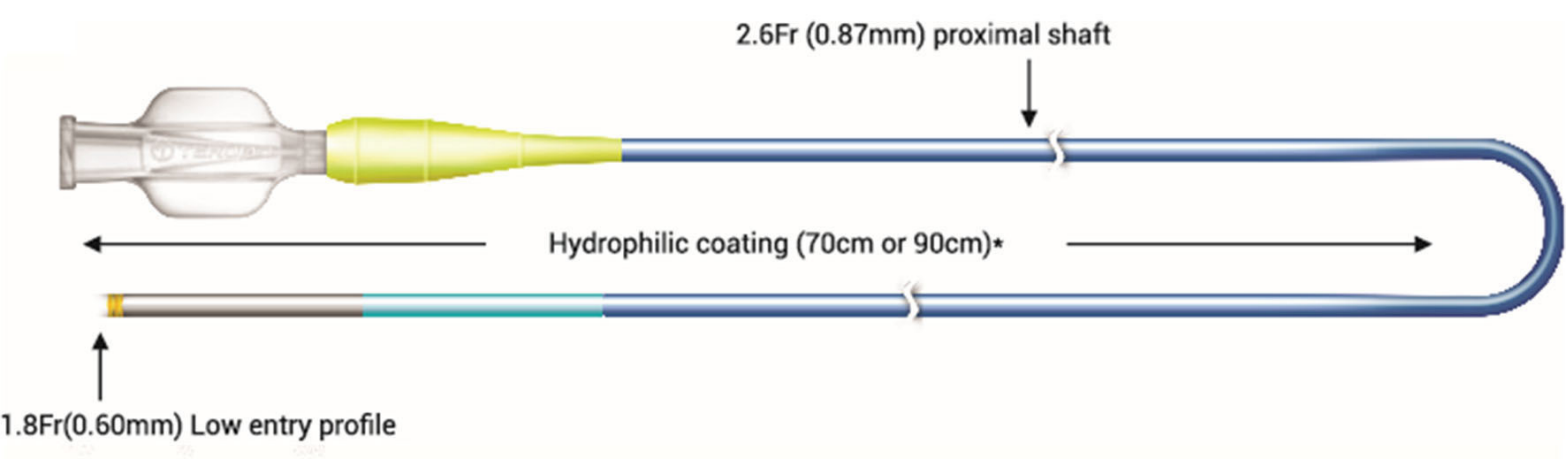
Single-lumen microcatheter (SLM) (Fine Cross^TM^ MG microcatheter) profile.

Single-lumen microcatheters (SLMs) are standard microcatheters intended to provide support to enable the placement of guidewires in a vessel. They are simple to handle and allow wire reshaping or wire exchange without losing the vessel entry. They improve the guidewire penetration ability and could prevent wire tip prolapse/plop (Figure 1). These microcatheters can also be maneuvered through a tortuous arterial segment proximal to a lesion by providing extra support to a guidewire. They can also be used to visualize a distal vessel by injecting a contrast medium (2). SLMs are preferred to over-the-wire (OTW) balloons in allowing an accurate visualization of their tip location with the availability of a marker at the tip, whereas small balloons (1.0–1.5 mm in diameter) have a marker in their mid-shaft and a distal location of the tip is not truly clear on fluoroscopy. FineCross™ MG (Terumo Interventional Systems, Tokyo, Japan), Caravel™ (Asahi Intecc Co., Ltd., Agayo, Japan), Mcath® (Acrostak, Winterthur, Switzerland), Tornus® (Asahi Intecc Co., Ltd., Agayo, Japan), and Corsair®/Corsair® Pro (Asahi Intecc Co., Ltd., Agayo, Japan) are some of the widely used single-lumen microcatheters in the Indian market at present (Table 1). Recently, Teleflex and Vascular solutions have received an approval from India for their products SuperCross® and Turnpike® microcatheter, respectively. Other microcatheters like MambaFlex® (Boston Scientific, Massachusetts, USA), Teleport® (Cardiovascular Systems, Inc., Minnesota, USA), and Microcross® (Roxwood Medical, Inc., California, USA) have not got approval in India at present.

**Table 1:** A brief comparative table outlining the specific mechanical features of various single-lumen microcatheters.

**Microcatheters**	**FineCross™ MG (Terumo Interventional Systems, Tokyo, Japan)**	**Caravel™ (Asahi Intecc. Co Ltd, Agayo, Japan)**	**Corsair^®^/ Corsair Pro^®^ (Asahi Intecc. Co Ltd, Agayo Japan)**	**SuperCross™ (Teleflex, North Carolina, USA)**	**Turnpike^®^ (Vascular solutions, Minneapolis, USA)**
Catheter length	130 and 150 cm	135 and 150 cm	135 and 150 cm	130 and 150 cm	135 and 150 cm
Distal outer dia	1.8 Fr (0.60 mm)	1.9 Fr (0.62 mm)	2.6 Fr (0.87 mm)	1.8 Fr (0.61 mm) and 2.4 Fr (0.79 mm)	2.2 Fr (0.74 mm) and 2.6 Fr (0.86 mm)
Distal inner dia	0.018” (0.45 mm)	0.016” (0.40 mm)	0.015” (0.38 mm)	0.017” (0.43 mm)	0.015” (0.38 mm)
Proximal outer dia	2.6 Fr (0.87 mm)	2.6 Fr (0.85 mm)	2.8 Fr (0.92 mm)	2.5 Fr (0.84 mm) and 3.2 Fr (1.07 mm)	2.9 Fr (0.97 mm) and 3.1 Fr (1.02 mm)
Proximal inner dia	0.021” (0.53 mm)	0.022” (0.55 mm)	0.018” (0.46 mm)	0.018” (0.46 mm) and 0.021” (0.53 mm)	0.021 (0.53 mm)
Radiopacity	Single golden marker located 0.7 mm from the tip	Yes	Yes	Yes	Yes
Coating	Hydrophilic (distal 70–90 cm)	Hydrophilic	Hydrophilic	Hydrophilic (distal 80 cm)	Hydrophilic (distal 60 cm)
Guidewire compatibility	0.014 (0.316 mm)	0.014” (0.316 mm)	0.014” (0.316 mm)	0.014” (0.316 mm)	0.014” (0.316 mm)

### Double-Lumen Microcatheters

**Figure 2:**
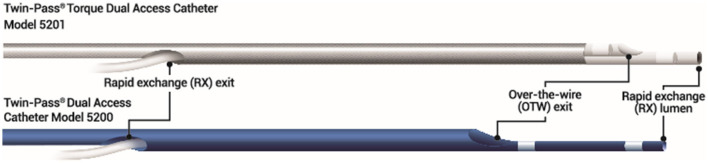
Double-lumen microcatheter (DLM) (Twin-Pass Dual® Access microcatheter) profile.

Double-lumen microcatheters (DLMs) have two lumens to facilitate precise and independent handling of two different guidewires. One lumen consists of a rapid delivery system, which has a distal port at the distal end of a catheter, and another lumen is OTW with the distal end opening slightly proximal to a distal tip by centimeter or so and runs the whole length of a catheter (Figure 2) with a proximal end at the proximal hub of a catheter. The two radiopaque markers are positioned in such a way to denote the exit ports of both lumens. Twin-Pass® (Teleflex, Wayne, USA), Sasuke® (Asahi Intecc Co Ltd., Akatsukicho, AIC, Japan), and Crusade® (Kaneka Medix Corp., TYO, Japan) are few DLMs available for clinical use in the Indian market. Other DLMs like NHancerRx® (IMDS, Roden, Netherlands), FineDuo® (Terumo Interventional Systems, Tokyo, Japan), and ReCross® (IMDS, Roden, Netherlands) have not got approval in India at present.

## Need for a Microcatheter and Its Selection Criteria

**Figure 3:**
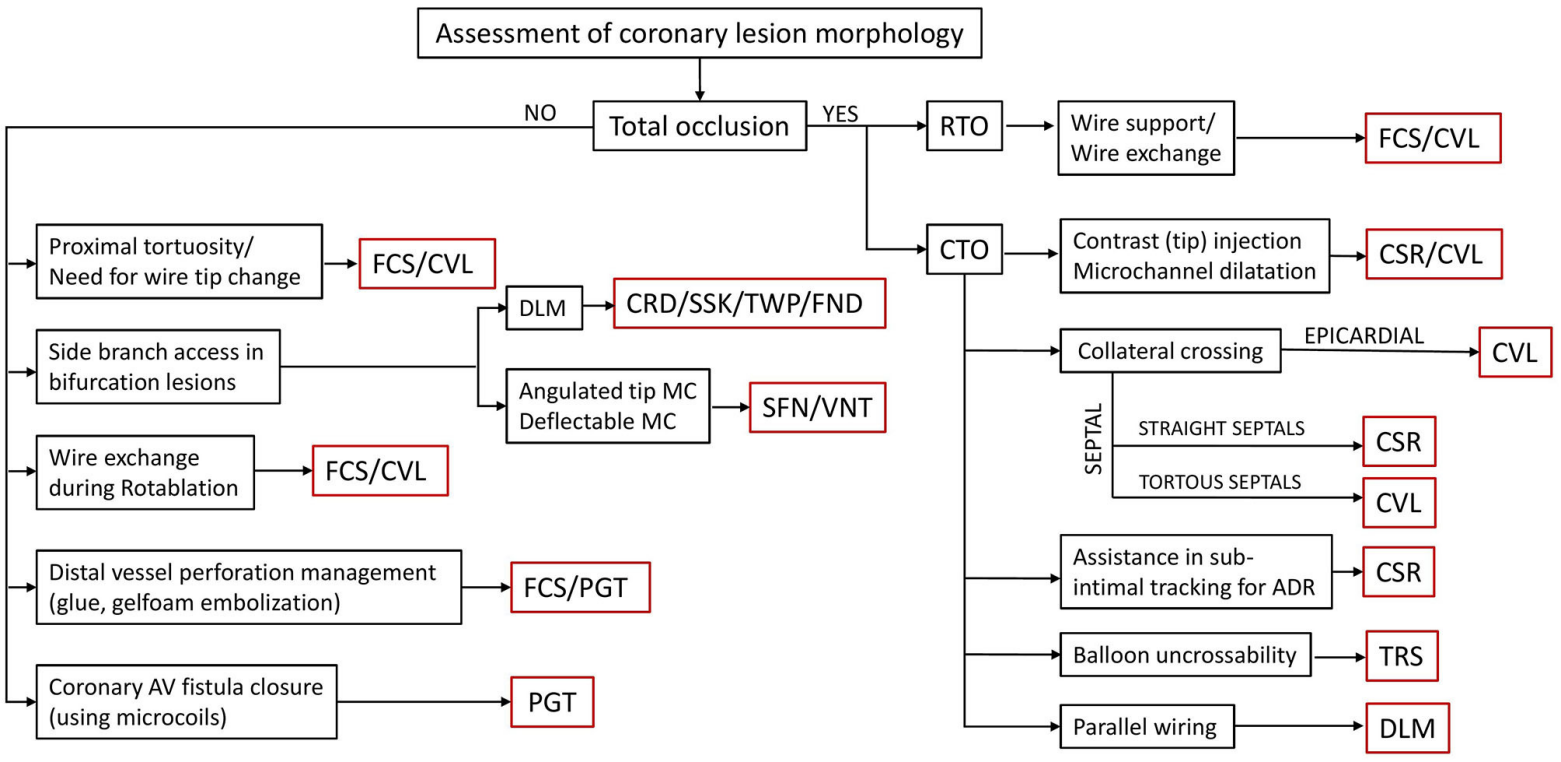
An algorithm for the selection of a microcatheter depending on the lesion characteristics and functional requirement for that particular coronary anatomy. MC, microcatheter; RTO, recent total occlusion; CTO, chronic total occlusion; FCS, Finecross; CVL, Caravel; CSR, Corsair; TRS, Tornus; DLM, dual-lumen microcatheter; CRD, Crusade; SSK, Sasuke; TWP, Twinpass; FND, Fineduo; SFN, SwiftNinja; VNT, Venture; PGT, Prograte.

This article primarily discusses an experience of the advisory board in the use of different SLMs during complex PCIs and the criteria used by them to decide on the need of a technique for the use of different microcatheters. An algorithm depicting the selection of a particular microcatheter according to the involvement of the relevant coronary anatomy is shown in Figure 3.

### Structural Properties/Characteristics of Microcatheters

As per the experts, multiple microcatheter features impact its selection *viz*. tip profile, shaft profile, tip and shaft flexibility, inner lumen diameter, radiopacity of the device, and microcatheter tube structure a.

#### Tip and Shaft Profile

- *Long vs. short taper tip*: The functionality of a microcatheter varies with the tip taper structure. An expert panel with its clinical experience opined that, for the crossability of long narrow channels like septal collaterals, microcatheters with a long-tapered tip are beneficial (like Caravel^TM^ or Corsair®/Corsair® Pro). Meanwhile, for better penetration and pushability like crossing a CTO lesion, a short-tapered tip is required (like FineCross™ MG). For instance, while maneuvering an ectatic vessel segment leading to a small channel tract, the operator needs microcatheters to be stable in the ectatic segment, and thus a short and flexible tapered tip catheter (like FineCross™ MG) is helpful in comparison to a long-tapered tip catheter (like Corsair® or Caravel™).
- *Tip diameter*: The distal and proximal end diameter of a tip taper also determines which microcatheter can be selected for an intervention. For example, when comparing the tip diameter, Corsair® has a smaller distal tip diameter than Caravel™ (1.3 vs. 1.4 Fr) but after the increment as we rise up the taper, the proximal end diameter for Caravel™ is smaller compared to Corsair® (1.9 vs. 2.6 Fr). Thus, maneuvering becomes difficult through narrow tracts due to a bigger shoulder of Corsair® at the proximal end of a taper. A distal outer diameter (tip end) provides compatibility with the vessel diameter that needs to be accessed. When an epicardial vessel, e.g., epicardial collateral or smaller branches, with a smaller diameter needs to be accessed, then microcatheters with a smaller tip diameter are the best (like FineCross™ MG with 1.8 Fr tip diameter). FineCross™ GT has an additional short length taper of 1.3 mm (with the tip diameter tapered downward from 1.8 to 1.7 Fr).
- *Wire diameter compatibility*: Inner lumen diameter provides the space for wire handling. Therefore, the selection of microcatheters should be such that the percutaneous transluminal coronary angioplasty (PTCA) wire must snugly fit within the lumen of microcatheters as per the specifications of the inner luminal diameter of microcatheters. For example, if a 0.018″ diameter lumen microcatheter is provided for a 0.014″ diameter wire, a ledge creating a “razor effect” could be produced between the wire and the tip of a microcatheter due to a gap between the microcatheter lumen and the wire, which could obstruct pushability. Reducing this gap will increase pushability, trackability, and support while maneuvering. However, if the wire diameter is too large, it will fight against the inner lumen wall, thus causing a friction between the wire and microcatheter inner lumen.
- *Angulated tip*: Angulated tip microcatheters have a specific degree (45°, 90°, and 120°) of short length bend at the hydrophilic tip end. This eases out the wiring of a side branch in bifurcated lesions. It also helps in negotiating extreme bends and proximal tortuosity in vessels having non-aorto ostial critical lesions at the bend. Steerable angulated tip microcatheters [SwiftNinja™ microcatheter (Merit Medical, West Jordan, UT, USA), Venture deflectable catheter (Teleflex, Wayne, USA)] additionally enable the successful *super-selective* cannulation and wiring of tortuous branches.

#### Radiopacity

One of the features for selecting microcatheter is their radiopacity or radiopaque marker near the tip, which helps in an accurate visualization of the tip location (3). Complete radiopacity (like in Corsair® or Caravel™) has its limitations when working on a distal end as it is difficult to assess the situation of a vessel at a proximal end.

#### Trackability

It is correlated with the force needed to reach the lesion and beyond by maneuvering through a tortuous trajectory, which needs to be as low as possible. For this, the construction of microcatheters needs to be very flexible or floppy and this can be achieved by choosing an appropriate polymeric material like a hydrophilic polymer or coating, polytetrafluoroethylene, polyurethane, etc. Hydrophilic coating in a microcatheter is also related to a rotational function rather than pushability alone (like FineCross™ MG), which might be necessary for a procedure (3).

### Clinical Applications in Coronary Interventional Procedures

**Figure 4:**
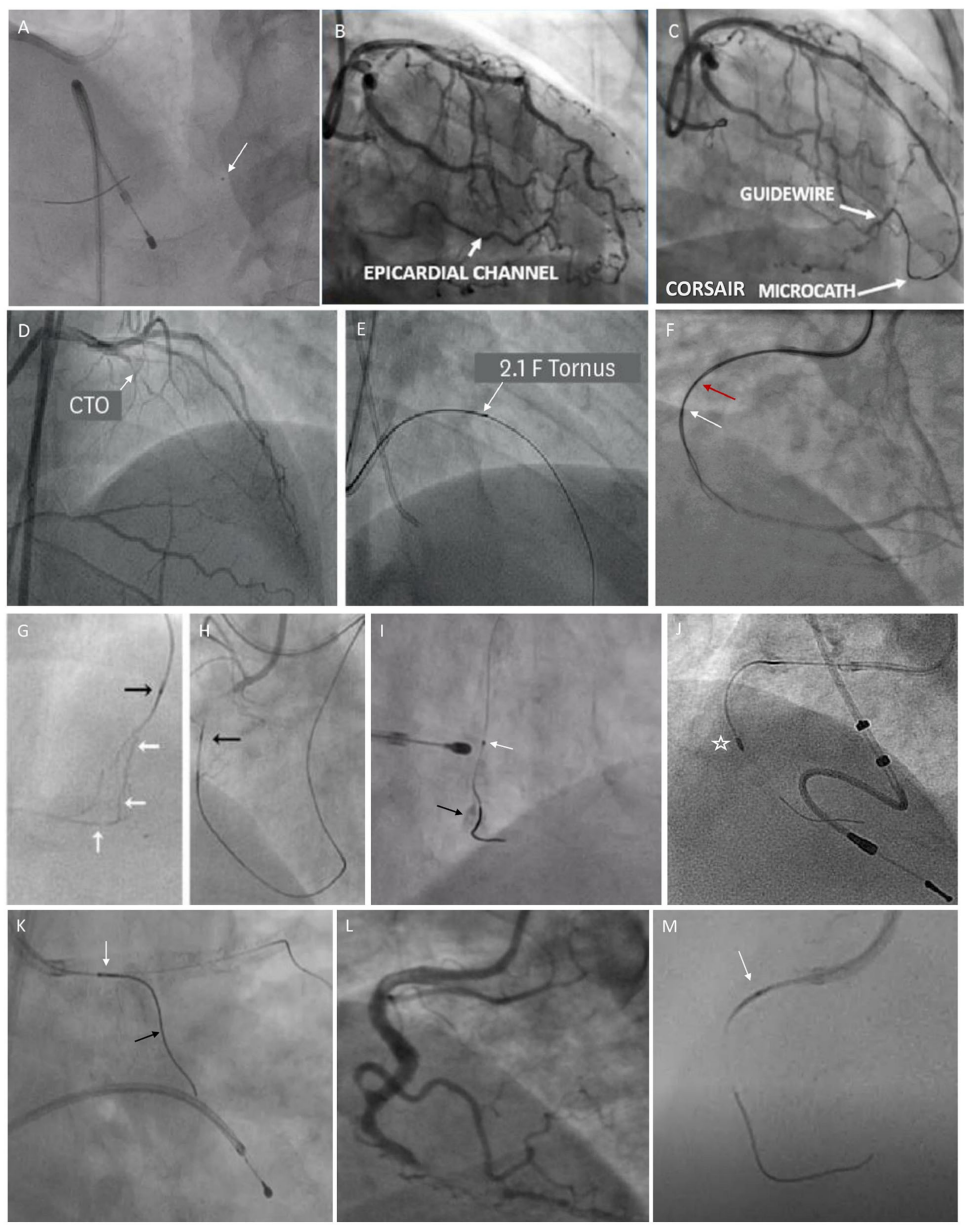
Fluoroscopic images illustrating the usage of various microcatheters in different scenarios. **(A)** shows Fine Cross microcatheter in distal right coronary artery (RCA) (white arrow) being used for wire exchange. **(B, C)** show an epicardial collateral from left anterior descending (LAD) to distal RCA, which is traversed by Corsair microcatheter. **(D, E)** show a mid LAD-CTO lesion being crossed by Tornus microcatheter (a white arrow). **(F)** shows a parallel wire technique using Crusade microcatheter for revascularizing RCA-CTO (a red arrow shows over-the-wire (OTW) lumen proximal marker and a white arrow shows a rapid exchange lumen distal marker). **(G)** shows tortuous LAD septal collaterals to distal RCA. **(H)** shows Corsair microcatheter (a black arrow) for dilating and crossing the septal channels (a white arrow). **(I)** shows that Caravel microcatheter (a white arrow) used for wire exchange enable Rotawire to cross into calcified distal RCA (a black arrow). **(J)** shows Rotablation burr (asterisk) for calcium debulking in RCA over Rotawire. **(K)** shows Sasuke double-lumen microcatheter (white arrow) facilitates the wiring of a side branch (a black arrow) while maintaining access to the main branch. **(L, M)** show Caravel microcatheter (white arrow) navigating the tortuous proximal RCA during CTO intervention.

The selection of microcatheters is also influenced by conditions like vessel anatomy (irregularity, tortuosity, angulations, bifurcations, and epicardial or septal collaterals), lesion location (distal, mid, or proximal), lesion morphology (anomalous ostial lesion, recent total occlusions (RTOs), etc.), CTO crossing strategy (antegrade vs. retrograde), and a guidewire to balloon crossability issues. The same has been illustrated using various fluoroscopic images in Figure 4.

#### CTO Lesion

In a CTO-PCI attempt *via* an antegrade or a retrograde approach, the use of a microcatheter is crucial in providing better support to the CTO wire, enhanced penetration capacity, reshaping of the guidewire tip, easy guidewire exchanges, the protection of a proximal vessel from stiff CTO guidewire-induced injuries (a proximal cap in case of antegrading and a distal cap in case of retrograding), and also facilitating guidewire maneuvering to side branches (in case of bifurcations in ostial circumflex CTOs) (5). The microcatheter further helps in stepping down to a regular workhorse wire after having crossed the CTO lesion, which is soft and flexible, provides an easier manipulation and gentler maneuvering in distal branches and avoid the perforation of distal vessels (6, 7). For CTO operators, a challenging part is the point when reentry is very tough and sometimes may obstruct a balloon from crossing over. Thus, instead of using a small low profile semi-compliant balloon, stiffer microcatheters (like Corsair®) can be used to dilate the false channel produced by the wire dissection into the subintimal space and create a passage for wire exchange facilitating the deployment of Stingray™ CTO system during an antegrade dissection reentry procedure.

#### RTO Lesion

As per the experts, microcatheters are also handy in dealing with RTOs. For example, a total occlusion which is 30-day old, regular guidewire (like Sion™ wire) may not be stiff enough to maneuver through a lesion. Meanwhile, the same wire in a microcatheter in such a situation increases wire support and assists lesion crossing. Moreover, it can also be exchanged for a hydrophilic polymer jacketed wire with increased tip load (e.g., Pilot 150 or Pilot 200) if the workhorse wire fails to cross the RTO lesion.

#### Interventional Epicardial or Septal Collaterals

The condition of vessel *i.e*., angle of entry, angle of donor vessel and angle at base of heart, if acute, is a significant barrier to both guidewire and microcatheter navigation (4). For treating epicardial vessels, operators prefer a flexible and tapered tip (like FineCross™ MG) that does not damage the vessels. Often during the intervention, the channels have to be stretched and dilated for facilitating the passage of microcatheters through a tortuous path and thus need the prevention of recoiling, which cannot be achieved by microcatheters with a short-tapered tip (e.g., FineCross™ MG). Caravel™ or Corsair® microcatheter can be used to circumvent these vessels and dilate the channels en route. While crossing a collateral, microcatheters can also be used to inject an contrast to visualize the finer channels (tip injection).

#### Angulated Lesion

During catheterization, operators often come across an anatomy, which requires a guidewire to have different curves for entering a branch and to cross a lesion. A microcatheter is vital in such situations enabling the access to a proximal vessel, then advancing a microcatheter and leaving it beyond the entry point of a vessel, replacing the wire with the required tip shape followed by balloon or stent delivery to perform angioplasty.

#### Bifurcated Lesion

If initially a side branch is inadvertently wired and an operator wishes to wire the distal main branch without losing access to a side branch, then a DLM comes in handy while successfully securing side branch accesses. The same could also be done *vice versa*.

#### Proximal Tortuosity

If a vessel is tortuous, then a stronger and stiffer microcatheter will not traverse the path. Meanwhile, if a microcatheter is more flexible, it will curl as a snake conforming to the path. Therefore, the flexibility of a distal part (5–10 cm from a tip) of microcatheters is important. If an operator is accessing tortuous anatomy, the preference of an operator will be to use a catheter like FineCross™ MG or Caravel^TM^ rather than the braided microcatheter Corsair®, which rotates while advancing as it is not advisable to use the rotation technique in tortuous anatomy. If a steerable catheter with the rotation function (like Ventura®, Teleflex, USA) is used, it will help in maneuvering through the difficult angled anatomy and increase support at the tip.

#### Calcified Lesions

As per the experts, if a proximal vessel trajectory is calcified and/or tortuous, it creates navigation issues while guiding and retracting, and may damage the microcatheter tip (like Caravel™ tip may break). For this purpose, the operator could use a regular wire inside a microcatheter (for example, FineCross™ MG which has a smaller diameter to maneuver) to reach the lesion followed by the replacement of this wire with another wire (e.g., atherectomy selective RotaWire^TM^) or device as per the needs of crossability of the lesion.

#### Balloon Uncrossable Lesions

During CTO-PCI, while treating critically stenosed vessels with a bend trajectory, the operator at times finds that the wire is crossed but it is difficult to cross the lesion with a balloon. Thus, initially improving wire support, which is done with a microcatheter, is at times helpful. In these situations, a Tornus® microcatheter, which moves forward with a corkscrew action of up to 20 counterclockwise turns, could work by crossing the lesion.

#### Guidewire-Related Coronary Tip Perforations

One of the microcatheter functions is to deliver microcoils to distal coronaries to seal a perforation. For operators, one of the challenges is to pair the preferred coil maximum diameter to microcatheters with an optimal inner lumen diameter. For example, a 0.018″ diameter coil can only be pushed through a microcatheter that has an inner lumen diameter of 0.018″ or greater (like Prograte®, Terumo Interventional Systems, Japan, which has 0.03–0.04″ in diameter).

## Conclusion

Interventional cardiologists use various lesion crossing strategies for PCI procedures coupled with microcatheters for optimal guidewire manipulations through the complex coronary anatomy. Microcatheter characteristics and structure are correlated to the type of functions it can perform during a complex PCI. Operators prefer a microcatheter, which can be easily maneuvered through tortuous or calcified lesion trajectories with tip flexibility, maintain wire tip integrity, enhance wire support, assist in wire exchanges, avoid vessel damage, and reduce procedural time. Thus, microcatheter selection and operating techniques are vital to a successful treatment plan, which should be based on the recommendations derived for clinical expertise and operator efficiency. For next-generation microcatheters, an increase in support without compromising on flexibility and lowering of the tip profile in comparison to the ones currently present in the market is needed.

## Author Contributions

PG conceptualization of study and reviewed and edited the final draft. AS prepared the initial draft and involved in editing the text, and tables and figures for the study. SK, SR, NS, PP, and DC provided valuable inputs during the discussion of study, reviewed the final draft of the study, and were involved in planning of the study. All authors contributed to the article and approved the submitted version.

## Funding

The authors declare that this study received funding from Terumo India Private Limited. The funder was not involved in the study design, collection, analysis, interpretation of data, the writing of this article or the decision to submit it for publication.

## Conflict of Interest

The authors declare that the research was conducted in the absence of any commercial or financial relationships that could be construed as a potential conflict of interest.

## Publisher's Note

All claims expressed in this article are solely those of the authors and do not necessarily represent those of their affiliated organizations, or those of the publisher, the editors and the reviewers. Any product that may be evaluated in this article, or claim that may be made by its manufacturer, is not guaranteed or endorsed by the publisher.

## References

[1] Estrada JR Paul JD Shah AP Nathan S. Overview of technical and cost considerations in complex percutaneous coronary intervention. Int Cardiol Rev. (2011) 9:17–22. 10.15420/icr.2011.9.1.17 29588772 PMC5808630

[2] Brilakis ES Mashayekhi K Tsuchikane E Abi Rafeh N Alaswad K Araya M. Guiding principles for chronic total occlusion percutaneous coronary intervention. Circulation. (2019) 140:420–33. 10.1161/CIRCULATIONAHA.119.039797 31356129

[3] Mishra S. Language of CTO interventions - focus on hardware. Indian Heart J. (2016) 68:450–63. 10.1016/j.ihj.2016.06.015 27543466 PMC4990808

[4] Dash D. A step-by-step guide to mastering retrograde coronary chronic total occlusion intervention in 2018: the author's perspective. Indian Heart J. (2018) 70:S446–55. 10.1016/j.ihj.2018.08.011 30595306 PMC6310897

[5] Vemmou E Nikolakopoulos I Xenogiannis I Megaly M Hall A Wang Y. Recent advances in microcatheter technology for the treatment of chronic total occlusions. Expert Rev Med Devices. (2019) 16:267–73. 10.1080/17434440.2019.1602039 30929525

[6] Thompson R. Isolated coronary ostial stenosis in women. J Am Coll Cardiol. (1986) 7:997–1003. 10.1016/S0735-1097(86)80217-0 3958382

[7] Lefort B Saint-Etienne C Soulé N Ma I Dion F Chantepie A. Perforation of the atretic pulmonary valve using chronic total occlusion (CTO) wire and coronary microcatheter. Congenit Heart Dis. (2019) 14:814–8. 10.1111/chd.12812 31290594

